# Does an Age-Specific Treatment Program Augment the Efficacy of a Cognitive-Behavioral Weight Loss Program in Adolescence and Young Adulthood? Results from a Controlled Study

**DOI:** 10.3390/nu11092053

**Published:** 2019-09-02

**Authors:** Petra Warschburger, Jana Zitzmann

**Affiliations:** Department of Psychology, University of Potsdam, Karl-Liebknecht-Str. 24–25, 14476 Potsdam, Germany

**Keywords:** emerging adults, adolescents, behavioral weight loss, obesity, controlled trial, quality of life

## Abstract

Research on weight-loss interventions in emerging adulthood is warranted. Therefore, a cognitive-behavioral group treatment (CBT), including development-specific topics for adolescents and young adults with obesity (YOUTH), was developed. In a controlled study, we compared the efficacy of this age-specific CBT group intervention to an age-unspecific CBT group delivered across ages in an inpatient setting. The primary outcome was body mass index standard deviation score (BMI-SDS) over the course of one year; secondary outcomes were health-related and disease-specific quality of life (QoL). 266 participants aged 16 to 21 years (65% females) were randomized. Intention-to-treat (ITT) and per-protocol analyses (PPA) were performed. For both group interventions, we observed significant and clinically relevant improvements in BMI-SDS and QoL over the course of time with small to large effect sizes. Contrary to our hypothesis, the age-specific intervention was not superior to the age-unspecific CBT-approach.

## 1. Introduction

The increasing prevalence of overweight and obese adolescents and young adults throughout the world marks an alarming development. Data from the representative Germany-wide KiGGS-studies (“Studie zur Gesundheit von Kindern und Jugendlichen in Deutschland”) showed that 16.2% of the girls and 18.5% of the boys aging from 14 to 17 years are overweight and even 7.7% and 9.2%, respectively, are obese [1]. Even higher rates were observed among young adults, whereby 30% of the females and 35.3% of the males aged 18 to 29 years were overweight [2]. The transition period from adolescence to adulthood especially, is characterized by rapid weight gain [3,4,5].

During emerging adulthood, a developmental period, which is often defined between 18 and 25 years of age [6], the social and physical environment significantly change and pose challenges to the adolescents. Besides, dealing with role transitions, balancing autonomy and responsibility mark central developmental tasks for young adults [6]. It can be assumed, that the unique characteristics of this sensitive transition period may contribute to establishing long-term health-related behavioral patterns [4]. It was shown that many unhealthy changes in physical activity patterns and diet practices, which are also related to the emergence of obesity, develop during emerging adulthood [4,7,8]. Therefore, this sensitive developmental stage is highly relevant for interventional efforts targeting weight gain and obesity.

Long-term consequences of obesity are alarming and diverse. On one side, high costs for the health care system occur [9,10]. On the other side, various consecutive symptoms emerging from obesity and the development of further risky behavior, which partly maintain obesity, are often observed—especially among adolescents and young adults. In this regard, research has not only demonstrated negative health consequences, such as cardiovascular morbidity and type 2 diabetes, but also severe psychological consequences [11,12]. Among adolescents, an adverse and persistent impact of obesity was found on several dimensions of the emotional well-being, such as body dissatisfaction, self-esteem and depression, as well as on further disordered eating behaviors [13,14,15]. Moreover, research indicated that weight-based stigmatization and teasing of overweight individuals is widespread, possibly explaining the mentioned negative obesity-related psychological outcomes [16]. Once manifested, obesity likely remains until adulthood [17,18] and hence increases the risk of developing chronically physiological, psychological and psychosocial comorbidities.

The health and social well-being of people with obesity clearly demonstrate the need for interventions, which not only aim at reducing weight but also at reaching a long-term stabilization of the achieved weight loss. Research on existing interventions targeting overweightness and obesity demonstrated that a combination of nutrition, physical activity and behavioral modification is necessary to achieve long-term success [19,20,21]. In this regard, inpatient rehabilitation constitutes an important pillar for the treatment of obesity in Germany [22]. This highly controlled and supportive environment is considered as a particular suitable treatment option for adolescents and adults with underlying comorbidities. Besides the importance of a multidisciplinary approach, previous research consistently indicated that contents and methodology should match the developmental status of the individuals and take age-related needs into account [3,15,20,23,24,25,26]. Therefore, targeted weight loss interventions and hence a development-sensitive approach seem crucial [27]. This is supported by a review from Laska and colleagues [26] who showed that existing interventions developed for adults are less effective when offered to adolescents and young adults (18 to 35 years of age).

However, most existing weight loss interventions fail to address these subgroup-specific aspects [23,28]. Evaluations of numerous interventions showed that adolescents from the age of 18 years are typically undergoing the same treatment as older adults. There are barely any age-appropriate adaptations, which take developmental tasks of emerging adults into account [23,26]. In a qualitative study, Warschburger [29] found out, that emerging adults prefer age-homogenous group concepts, considering development specific challenges. Besides issues concerning the program content, most intervention studies addressed either children of younger age (<12 years) or adults, failing to evaluate the efficacy of interventions for adolescents in transition to adulthood. In a recent review by Thomason and colleagues [21], only eight randomized-controlled trials (RCTs) with adolescents were identified between 2008 and 2014, whereby most of the RCTs concentrated on adolescents with mean ages ranging from 13 to 15 years. Those conclusions were confirmed by Boff and colleagues [30] in their review as well.

In the last years, first weight loss interventions adapted for emerging adulthood were developed, showing mainly promising results. Gokee-LaRose and colleagues [24] adapted evidence-based behavioral weight loss programs for emerging adults by considering psychological needs outlined by Self-Determination Theory and found beneficial effects regarding weight loss. Emerging adults participating in this web-based 3-month lifestyle intervention with optional community sessions tended to lose more weight than those participating in web-based or face-to-face interventions approximated to adult weight loss programs (*f*_post treatment_ = 0.27, *f*_3-month follow-up_ = 0.33; [24]). Furthermore, several brief lifestyle interventions developed to promote frequent self-weighting were effective in preventing age-related weight gain and showed advantages over age-unspecific interventions (e.g., [31,32]).

Although there is a common agreement that psychosocial outcomes such as Quality of Life (QoL) should be considered as well [33]; studies investigating psychosocial outcomes are rare. In their meta-analysis, Ligthart and colleagues [34] included 11 studies examining the effects of multidisciplinary intervention programs compared with control interventions for overweight and obese children (three to 18 years of age) on QoL. Besides a nonsignificant long-term trend, no advantage of multidisciplinary intervention programs was observed. However, beneficial improvements in QoL among children and adolescents (8 to 19 years of age) participating in an intensive lifestyle treatment were observed by different researchers [35,36]. Furthermore, Hoedjes and colleagues [35] observed a positive relationship between weight loss and improvements in physical QoL domains. To our knowledge, only one study [37] analyzed the intervention effects on QoL among emerging adults (14 to 25 years of age). By comparing a structured, manual-based intervention focusing on coping with obesity and its acceptance with treatment as usual (focusing on weight loss), they found no group differences regarding QoL, but observed explorative evidence for a promising effect on social exclusion. Since previous studies show great heterogeneity in terms of program content and treatment duration, definitive conclusions regarding the effect of age-specificity cannot yet be drawn.

To summarize, research shows that there is a lack of evaluated approaches targeting adolescents and young adults, especially when it comes to development-sensitive interventions. Furthermore, the effectiveness of existing intervention programs seems to be disappointing when applied to emerging adults [26]. Based on previously reported evidence, the consideration of age-specific developmental challenges and tasks might help to improve the effectiveness of current interventional approaches. Therefore, a health educational group treatment, based on a cognitive-behavioral background, which includes development-specific topics for adolescents and young adults with obesity aged 16 to 21 years was developed and evaluated in a controlled study (the so-called YOUTH-study; [29]). We selected this age group, as in Germany, children and young adults up to the age of 21 can be referred to an inpatient rehabilitation clinic specialized in the treatment of children and adolescents. From the age of 21, they have to be referred to a clinic for adults. We compared its efficacy in an inpatient setting because these institutions provide a high standard of multidisciplinary treatment and cluster-randomized assignment can be guaranteed. Our main research question was whether an age-specific intervention augments the efficacy of a usual, state-of-the-art cognitive-behavioral treatment (CBT) that is provided across age-groups during a comprehensive inpatient treatment. As the YOUTH-intervention focuses on maintaining initial weight loss, we hypothesized that differences between both treatments in favor of the YOUTH-intervention will only become apparent at the 6- and 12-month follow-up. Our secondary research aim was to investigate overall treatment effects over the course of one year.

## 2. Materials and Methods

The study was approved by the ethics committee of the University of Potsdam at 18 April 2011 (#6/2011) and was conducted in line with the Declaration of Helsinki. Written informed consent for inclusion was provided by all participants and if minors from their parents before they participated in the study. Participating study centers and investigators are fully listed in the German Register of Clinical Studies (DRKS 00003424).

### 2.1. Participants

Adolescents and young adults with obesity (body mass index (BMI) > 97th percentile, respective BMI > 30 with accompanying diseases), aged 16 to 21 years, seeking weight loss treatment were recruited from seven rehabilitation clinics throughout Germany. All incoming registrations were screened for eligibility: At the beginning of the inpatient stay, all patients were seen by a medical doctor and a psychologist. For patients with suspected mental or intellectual disorders, standardized assessments (diagnostic interviews and/or questionnaires) were carried out in order to evaluate the eligibility. Participants with inadequate language skills, with severe cognitive impairments, or with secondary causes of obesity were excluded.

### 2.2. Procedure

**Figure 1:**
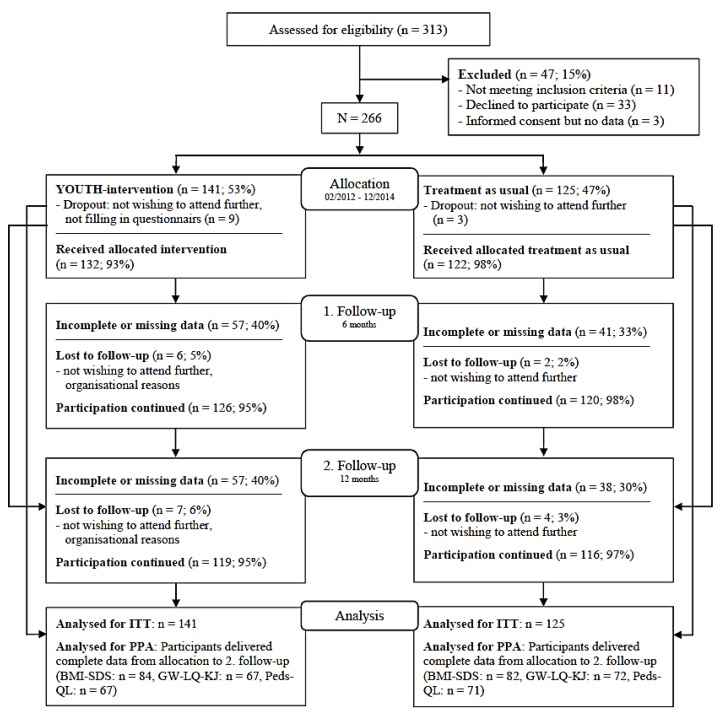
Study flow chart.

Of the 313 participants originally screened, 266 met the inclusion criteria (see Figure 1). Depending on their date of arrival in the inpatient clinic, we allocated the participants either to the treatment as usual (TAU; *n* = 125), as control condition, or the YOUTH-intervention (*n* = 141). We refrained from completely randomizing the participants in order to avoid spill-over effects in the clinical setting. Therefore, data collection started with the TAU and was followed by the YOUTH intervention group (IG). All measurements were taken at four times: Immediately at the beginning (T1) and at the end (T2) of the intervention, as well as 6 (T3) and 12 (T4) months after the end of the rehabilitation stay.

### 2.3. Interventions

**Common features.** The IG and TAU were comparable in all general treatment services offered for adolescents and young adults in a quality assured multidisciplinary lifestyle intervention (e.g., nutrition counselling, physical activity program, and leisure activities). In general, the rehabilitation stays lasted around 5 to 6 weeks (*M* = 39.21 days, *SD* = 18.28; with the opportunity to extend it upon reasonable request). Besides nutrition education and scheduled activity sessions, cognitive-behaviorally oriented group interventions were applied. In addition, some of the clinics offered special vocational consultations provided by the job center.

**Intervention program YOUTH.** The YOUTH-program was conceptualized as an age-specific group intervention program for adolescents and young adults with obesity. Based on both a qualitative and a quantitative survey examining the needs of the target group [29], the program content and methodological approach were compiled. The program was pilot-tested in a small group and discussed with several experienced practitioners in that field. The manualized program encompassed 9 sessions (see Table 1). It was delivered once or twice a week and carried out in closed groups (3–8 participants each group, 90-min sessions), only open for obese adolescents aged 16 years or older. Before the implementation of the intervention, we conducted a two-day train-the-trainer seminar. While the CBT sessions were conducted by experienced psychologists or educationalists, sessions referring to nutritional contents were supported by experienced nutritionists. 

Regarding the program content, attention is paid to ensure a direct relevance to everyday life of adolescents and young adults with obesity. Therefore, the YOUTH-intervention covers different topics such as diet, eating behaviors, stress management, problem-solving, interaction with the parents, asking for social support, school and profession, social competence and dealing with potential relapse. With a particular emphasis on the enhancement of self-esteem and self-management skills as important individual resources, we aimed to promote long-term treatment success (i.e., stabilization of the attained weight loss during rehabilitation). Therefore, cognitive-behavioral principles, such as goal-setting, self-monitoring, cue-control and reinforcement strategies were delivered through psychoeducation, group discussion, video-material, role-playing, individualized worksheets and “home-work” assignments. In addition, the YOUTH-program explicitly focuses on building up respective strengthening the motivation for change. After each lesson, participants rated on a 6-point Likert scale (from 1 ‘not important/useful’ to 6 ‘very important/useful’) their satisfaction with the intervention. Overall, participants reported a good satisfaction with the YOUTH-intervention (*M* = 4.18; *SD* = 0.85).

**Control condition TAU.** In contrast to the IG, TAU participants took part in an intervention program that was not specially designed for this age group and was delivered across age groups (i.e., younger children and older adolescents were trained together, and all participants received the same age-unspecific education material). Due to organizational reasons, those age-heterogeneous groups (e.g., with children from the age of 12 and adolescents up to the age of 18 or 21) are a common practice in Germany. The concrete education materials slightly differed from clinic to clinic, but all programs pursued the same objective—the building up of self-management skills. Comparable to the YOUTH-intervention, education of nutrition and eating behavior was provided, whereas no special focus was laid on adolescent-specific issues, such as autonomy from the parents, how to cope with (future) job applications or self-management via consequent goal pursuit. 

### 2.4. Measures

**Demographic data.** Demographic data including age, sex, perceived financial security and education were provided by self-report.

**Main outcome: BMI standard deviation score (BMI-SDS).** Based on height and weight, measured with standardized equipment, the participants’ BMI (kg/m^2^) was calculated and converted in the so-called BMI-SDS according to current German reference data [38]. The BMI-SDS adjusts for age- and gender-related physical changes in BMI. In accordance with national guidelines recommended by Kromeyer-Hauschild and colleagues [38], weight categories were defined as obese (BMI > 97th percentile) and severely obese (BMI > 99.5th percentile).

Height- and weight-data were assessed pre- (T1) and post-intervention (T2) by study nurses. At short-term (T3) and long-term (T4) follow-up, data were assessed by the attending general physicians at home (first choice) or when this was not possible in local pharmacies. Physicians received monetary reimbursement. All assessors were blind to group-assignments and study goals. Several reminders by post and telephone, as well as a reimbursement for the participants’ efforts were applied in order to minimize attrition bias. Additionally, height and weight data were assessed at all time points via self-report. 

**Secondary outcome: QoL.** The secondary outcome, QoL, was assessed with a generic and a disease-specific questionnaire. The adolescents’ and young adults’ weight-related QoL was assessed via the weight-specific quality of life questionnaire for overweight and obese children and adolescents (GW-LQ-KJ; [39]). The original questionnaire consists of 11 items (e.g., “I was dissatisfied with my figure because of my weight.”), rated on a 5-point Likert scale (from 1 ‘never’ to 5 ‘always’). For this study, we added two items addressing social aspects (“Because of my weight, I preferred to be alone rather than with others,” and “Because of my weight, I got the feeling of being stared at.”). The GW-LQ-KJ has good internal consistency (*α* = 0.87; [39]), which was confirmed by our data (*α* = 0.89). Furthermore, significant positive correlations (*r* between 0.27 and 0.56) of the GW-LQ-KJ with all scales of the Child Health Questionnaire (CHQ-87; [40]) supported its validity [39]. 

To evaluate generic aspects of the participants’ QoL, three subscales (emotional, social and school functioning) of the Pediatric Quality of Life Inventory (Peds-QL; [41]) were applied. Each subscale consists of 5 items (e.g., “I felt afraid or scared,” and “I found it hard to concentrate.”), rated on a 5-point Likert scale (from 1 ‘never’ to 5 ‘always’). The Peds-QL marks a reliable and valid questionnaire for children and adolescents with chronic diseases [41,42]. In line with previous evaluations (*α*_emotional_ = 0.73; *α*_social_ = 0.71; *α*_school_ = 0.68; [41]), a good internal consistency was confirmed by our data (*α*_emotional_ = 0.80; *α*_social_ = 0.87; *α*_school_ = 0.72). 

According to the procedure of Varni and colleagues [41], the raw values of the GW-LQ-KJ and the Peds-QL were transformed into values ranging from 0 to 100. The correlation between the total scores of both questionnaires was high (*r*_T1_ = 0.590; *r*_T2_ =.589; *r*_T3_ = 0.675; *r*_T4_ = 0.576; all *p* < 0.001). 

### 2.5. Data Analysis

All statistical analyses were performed using SPSS 23. Differences in baseline variables between the IG and the TAU were tested using independent t-tests (continuous variables) or chi-square tests (categorical variables). Intention-to-treat (ITT) analyses, including all participants initially allocated to one of the intervention groups and per-protocol analyses (PPA), as completer analyses, were performed. For PPA, participants with no or irregular participation within the intervention were excluded. From the 266 participants in the beginning, 235 continuously participated until the end of the study. However, some participants only provided data on the 6- or 12-month follow-up (see Figure 1). For analyses on BMI-SDS, objective weight data were used when available (*n* = 101). As objective and subjective weight data showed high significant correlations over all time points (*r* between 0.991 and 0.919, all *p* > 0.001), missing objective data were replaced by self-reported data. Besides, for ITT analyses, missing data within the outcome variables were replaced by simple imputation (mean change in the corresponding clinic, in regard to age and sex). For PPA, participants with missing data on the respective outcome variables at any time point were excluded. Additionally, we conducted sensitivity analyses by replicating the analyses described below, while successively leaving one of the rehabilitation clinics out.

For our main research question, group differences in BMI-SDS and QoL were tested at the 6-month follow-up (T3, short-term effect) and at the 12-month follow-up (T4, long-term effect) by analyses of covariance (ANCOVAs), controlling for baseline values (T1). Mean differences (*MD*) are reported to compare the IG with the TAU (negative values represent higher scores in the IG). Due to incomplete data sets, short- and long-term effects were analyzed separately. To report on the general efficacy of the treatment approaches over time, clinically relevant changes in BMI-SDS and QoL were reported descriptively. For BMI-SDS, a reduction of ≥ 0.2 BMI-SDS points can be considered as a clinically relevant treatment effect, and a reduction of ≥ 0.5 BMI-SDS points as a large treatment effect. For Peds-QL, Varni and colleagues [43] suggested that an increase of ≥ 4.4 scale points in the total scale score marks a minimal clinically meaningful difference. We adopted this cut-off for the Peds-QL subscales and the GW-LQ-KJ. Additionally, repeated measures analyses of variance (ANOVAs) and multivariate analyses of variance (MANOVAs) were calculated in order to examine changes in BMI-SDS and QoL over the four measurement points. In case of significant main effects, post hoc tests with Bonferroni correction were performed. *MDs* are reported to compare the previous with the later measurement points (negative values represent improvements over time). In additional explorative analyses, correlations (Pearson product-moment correlation) were used to examine associations between changes in BMI-SDS and time-correspondent changes in QoL. All statistical tests were performed at the 0.05 level of significance. 

## 3. Results

The results are reported separately for respective short- and long-term effects of treatment allocation and for changes over time. Each paragraph shows the results for BMI-SDS first, followed by the results for GW-LQ-KJ and Peds-QL. Besides results of ITT analyses based on data of 266 participants, results derived from a PPA are reported. For PPA, only participants who delivered complete data over all time points were included (BMI-SDS—*n* = 166; GW-LQ-KJ—*n* = 139; Peds-QL—*n* = 138).

### 3.1. Participant Characteristics at Baseline

In total, 266 participants (174 females) were included (mean age = 17.49 years, standard deviation age = 1.12). Participants’ characteristics are depicted in Table 2. Participants in the IG were significantly older than in the TAU (*t* = −2.285, *df* = 264, *p* = 0.023). At baseline, the groups neither differed in BMI-SDS (*t* = 0.488, *df* = 263, *p* = 0.626), nor in QoL (GW-LQ-KJ: *t* = 0.864, *df* = 263.92, *p* = 0.388; Peds-QLemotional: *t* = 1.684, *df* = 264, *p* = 0.093; Peds-QLsocial: *t* = 1.096, *df* = 264, *p* = 0.274; Peds-QLschool: *t* = 1.106, *df* = 264, *p* = 0.270).

### 3.2. Short-and Long-Term Effects of Treatment Allocation

For BMI-SDS, no group differences could be found, either at the 6-month, or at the 12-month follow-up. Baseline values of BMI-SDS had a significant effect on short-term, and long-term results (see Table 3). Similarly, for GW-QL-KJ, we observed no differences between groups for either short-term, or long-term effects. Again, baseline values of GW-QL-KJ showed significant effects on short-term, and long-term effects (see Table 3). Finally, regarding the Peds-QL domains emotional, social and school functioning, no differences between groups were observed for either short-term, or long-term effects. However, baseline values of the respective Peds-QL domains significantly influenced short-term, and long-term effects (see Table 3).

In sensitivity analyses, the previously mentioned results for BMI-SDS and GW-QL-KJ were corroborated. Slightly different results only emerged for the Peds-QL domain school functioning: We observed a significant group effect at 6-month follow-up time, with participants in the IG reporting higher values than in the TAU, significantly (*p* = 0.019), when leaving the participants (*n* = 32) of a small clinic out. When excluding the participants of a bigger clinic (*n* = 97) from the analyses, a significant group effect at 12-month follow-up was observed (*p* = 0.044), with participants in the TAU reporting higher values than in the IG.

### 3.3. Changes over Time

76.3% of the participants successfully reduced their weight during the inpatient treatment, with 10.5% achieving even large weight reductions. 22.9% showed only small improvements (< 0.2 BMI-SDS points). Only 0.8% had worsened compared to the beginning of the intervention. On average, participants showed a reduction of 0.32 (range: −1.36 to 0.15) BMI-SDS points during inpatient treatment. At T3, 44.8% of the participants had successfully sustained their clinically relevant weight loss since the end of the intervention, with an average reduction of further a 0.20 (range: −1.61 to 2.55) BMI-SDS points. Among those, 20.7% showed even large improvements. However, 29.3% had worsened compared to post intervention. While 37.6% of the participants were able to sustain their clinically relevant weight reduction at T4, 22.6% showed only small improvements (< 0.2 BMI-SDS points) and 39.8% had worsened compared to post intervention. On average, they lost further 0.13 (range: −1.49 to 1.24) BMI-SDS points since the end of the intervention.

Results of repeated measures ANOVA showed a significant, primary effect of time (ITT: *F* (1.98, 522.13) = 179.95, *p* < 0.001, *partial η^2^* = 0.405). Post hoc tests with Bonferroni correction showed significant reductions of BMI-SDS from T1 to T2 (*MD* = −0.32, *SE* = 0.01, *p* < 0.001), from T1 to T3 (*MD* = −0.52, *SE* = 0.03, *p* < 0.001), from T1 to T4 (*MD* = −0.45, *SE* = 0.03, *p* < 0.001), from T2 to T3 (*MD* = −0.20, *SE* = 0.03, *p* < 0.001) and from T2 to T4 (*MD* = −0.13, *SE* = 0.03, *p* < 0.001). However, from T3 to T4, BMI-SDS significantly increased (*MD* = 0.07, *SE* = 0.02, *p* = 0.021). Means and standard deviation scores are depicted in Table 4. For a PPA including 166 participants who completely reported weight data over all time points led to similar results: We observed a significant, primary effect of time (*F* (1.91, 313.42) = 106.40, *p* < 0.001, *partial η^2^* = 0.393).

Regarding the GW-LQ-KJ, 71.4% of the participants reported clinically significant improvements during the inpatient treatment, with an average increase of 15.02 scale points (range: −46.15 to 67.31). For the emotional domain of the Peds-QL, 43.6% of the participants reported clinically significant improvements during the inpatient treatment. Even higher proportions were observed for the social- (59%), and the school- (60.5%) related domains of the Peds-QL. At T3, 42.1% of the participants had successfully sustained their clinically significant improvement in GW-LQ-KJ; respectively, 43.2% in the emotional-, 36.8% in the social-, and 28.2% in the school-related domains of Peds-QL. However, a similar or even higher proportion of participants reported reductions in QoL since the end of the intervention (GW-LQ-KJ: 41.7%; Peds-QL domains emotional: 41.0%, social: 39.1%, school: 58.3%). At T4, only 36.1% of the participants had successfully sustained their clinically significant improvements in GW-LQ-KJ since the end of the intervention; respectively, 35.0% in the emotional-, 33.5% in the social-, and 27.8% in the school-related domains of Peds-QL. Again, a high proportion reported a decreasing QoL (GW-LQ-KJ: 55.3%; Peds-QL domains emotional: 54.1%, social: 45.5%, school: 60.9%).

For weight-related QoL, results of repeated measures ANOVAs showed a significant, primary effect of time (ITT: *F* (2.76, 727.75) = 87.03, *p* < 0.001, *partial η^2^* = 0.248; PPA: *F* (2.80, 383.56) = 42.44, *p* < 0.001, *partial η^2^* = 0.237). Again, post hoc tests with Bonferroni correction showed that scores significantly increased from T1 to T2 (ITT: *MD* = 15.11, *SE* = 1.16, *p* < 0.001), from T1 to T3 (ITT: *MD* = 18.44, *SE* = 1.31, *p* < 0.001), and from T1 to T4 (ITT: *MD* = 14.22, *SE* = 1.33, *p* < 0.001). No significant differences were observed between T2 and T3, nor between T2 and T4 (ITT: all *p* > 0.05). From T3 to T4, scores significantly decreased (ITT: *MD* = −4.22, *SE* = 1.05, *p* < 0.001). Means and standard deviation scores are depicted in Table 4.

Finally, for the generic QoL, results of repeated measures MANOVAs showed a significant, primary effect of time for all domains: Emotional (ITT: *F* (2.61, 690.07) = 5.59, *p* = 0.002, *partial η^2^* = 0.021; PPA: *F* (2.69, 366.36) = 3.03, *p* = 0.035, *partial η^2^* = 0.022), social (ITT: *F* (2.59, 682.52) = 26.75, *p* < 0.001, *partial η^2^* = 0.092; PPA: *n* = 138, *F* (2.73, 371.33) = 14.81, *p* < 0.001, *partial η^2^* = 0.098) and school-related QoL (ITT: *F* (2.78, 733.91) = 17.48, *p* < 0.001, *partial η^2^* = 0.062; PPA: *n* = 138, *F* (2.80, 381.24) = 10.34, *p* < 0.001, *partial η^2^* = 0.071). While post hoc tests showed significant improvements in the emotional domain from T1 to T3 (ITT: *MD* = 4.99, *SE* = 1.39, *p* = 0.002), scores significantly decreased from T3 to T4 (ITT: *MD* = −3.50, *SE* = 1.03, *p* = 0.005). For the social domain, post hoc tests showed significant score improvements from T1 to T2 (ITT: *MD* = 8.03, *SE* = 1.25, *p* < 0.001), from T1 to T3 (ITT: *MD* = 9.82, *SE* = 1.29, *p* < 0.001), and from T1 to T4 (ITT: *MD* = 7.87, *SE* = 1.35, *p* < 0.001). Furthermore, post hoc tests showed that scores of the school domain significantly improved from T1 to T2 (ITT: *MD* = 8.22, *SE* = 1.22, *p* < 0.001), from T1 to T3 (ITT: *MD* = 4.52, *SE* = 1.24, *p* = 0.001). From T2 to T3 (ITT: *MD* = −3.71, *SE* = 1.16, *p* = 0.009) and from T2 to T4 (ITT: *MD* = −5.46, *SE* = 1.20, *p* < 0.001), scores significantly decreased. Other differences showed no significance (ITT: all *p* > 0.05). Means and standard deviation scores are depicted in Table 4.

In additional exploratory analyses, we observed significant correlations between changes in BMI-SDS from T2 to T3, and corresponding changes in GW-LQ-KJ (*r* = −0.183, *p* = 0.001), as well as in the emotional domain of Peds-QL (*r* = −0.119, *p* = 0.035). Similarly, changes in BMI-SDS from T2 to T4 were significantly associated to corresponding changes in GW-LQ-KJ (*r* = −0.163, *p* = 0.004). Other correlation coefficients yielded no significance (all *p* > 0.05).

Again, the previous mentioned results for BMI-SDS, GW-QL-KJ and all Peds-QL domains could be replicated in sensitivity analyses.

## 4. Discussion

The main goal of our cluster-randomized controlled-study was to examine whether an age-specific cognitive-behavioral intervention augments the efficacy of a comprehensive age-unspecific intervention in terms of weight loss and QoL among adolescents and young adults with obesity. Our secondary research aim was to investigate overall treatment effects over the course of one year. Taken together, we observed significant and clinically relevant improvements in weight status and QoL over time, but contrary to our primary hypothesis, no indication of a superior efficacy of an age-specific intervention was found.

### 4.1. Short- and Long-Term Effects of Treatment Allocation

Contrary to our primary hypothesis, no significant differences between the efficacies of the two CBT modalities could be observed, either for weight loss, or for QoL. While Gokee-LaRose and colleagues [24] found promising effects of an age-adopted intervention compared to an age-unspecific treatment in an ambulatory setting, our data derived from an inpatient setting suggest that an age-specific approach cannot augment the efficacy of the comprehensive residential weight loss intervention in terms of weight loss and QoL. Regarding QoL, we are not aware of any study investigating the effect of age-specific interventions compared to TAU. Mühlig and colleagues [37] compared multilevel interventions with TAU among adolescents and young adults and observed no significant difference in QoL.

One explanation for this observation might be that, especially within inpatient settings, comprehensive intervention approaches combining diet modification, an increase in physical activity, stress reduction and a comprehensive cognitive-behavioral intervention are realized. Such life-style approaches are in correspondence with current treatment recommendations and previous studies have supported their efficacy [19,20,21,30,44]. Compared to the general efficacy [45] of the already existing multidisciplinary and intensive treatment approach applied in inpatient rehabilitation clinics, which was also confirmed by our results, the two different weekly CBT-sessions may have only made a small additional contribution. Hence, one might conclude that within this particular setting, the implementation of an age-specific outline of the cognitive-behavioral component of the complex intervention was not a crucial factor for the observed intervention effects and does not add substantial gain in terms of weight loss and QoL.

The more promising findings of age-specific interventions in the ambulatory setting [24] might be due to the general lower weight reductions [46] and smaller improvements regarding QoL [47] within ambulatory interventions as compared to inpatient treatment, reported in recent meta-analyses. As all participants showed favorable and quite large treatment effects, it was hard to identify the potentially existent small benefits of an age-specific adaptation within the less remaining variance. 

However, it might also be that an existing advantage of the YOUTH-program over the TAU might have not been detected because of methodological issues. The realization of our study within an inpatient setting is a double-edged sword. On one hand, as previously reported, several factors can be controlled more than in an ambulatory setting. On the other hand, it was not possible to completely control for a potential add-on due to the YOUTH-program. During their stay, the adolescents and young adults had various opportunities to learn from, and exchange experience with their age-mates: They shared their leisure activities and meal times in age homogenous housing groups across different conditions. Consequently, a social exchange even outside the therapy sessions was probable. Furthermore, both cognitive-behavioral group interventions were conducted by experienced health professionals, and all clinics were keen to realize age-homogenous groups. 

Another explanation for the non-significant differences at follow-up might be that the residential inpatient treatment far from home, and the unusual social environment, impeded the transfer to everyday life. Although the YOUTH-intervention focusses on the maintenance of initial weight loss, incorporating self-regulation strategies, such as self-monitoring and goal setting for the time after the rehabilitation, these strategies were not sufficient to yield superior effects compared to TAU. It seems crucial to continuously support and stimulate the weight loss respective maintenance efforts of the adolescents and young adults. Booster sessions or smart-phone applications might be a promising approach [47,48,49,50]. 

While we observed no difference between the age-specific YOUTH-intervention and the TAU in regard of weight reduction or QoL, the participants’ BMI-SDS respective QoL at baseline were significantly associated with the short- as well as the long-term treatment success. Regarding BMI-SDS, similar observations in inpatient settings were made by Hoffmeister and colleagues [45].

### 4.2. Changes over Time

In terms of weight loss during an intensive inpatient weight loss program, our results are comparable with, and partly even more promising than other reports from the literature among older children and adolescents [36,51,52]. Furthermore, overall changes in BMI compare favorably to those reported in recent meta-analyses among children and adolescents up to 19 years old [46,49,53], as well as among adults older than 18 years old [54]. In general, adolescents who participated in the inpatient treatment had lost 0.32 BMI-SDS points post treatment, 0.52 BMI-SDS points at the 6-month follow-up and 0.45 points at the 12-month follow-up. Considering emerging adults, our results are especially promising, as Zolotarjova and colleagues [47] reported less weight loss among older children and adolescents compared to younger children in their recent review. However, in two recent meta-analyses, no consistent age-specific differences in weight loss have been reported [46,49]. Similarly, Gokee-LaRose and colleagues [23] observed no statistically significant difference in weight loss between young and older adults participating in an outpatient behavioral weight loss program, when controlling for session attendance or treatment completion. Although, overall, we observed a slight weight gain (0.07 BMI-SDS points) from 6-month to one-year follow-up; 60.2% of the participants continued reducing their weight one year after the end of the intervention. These data are especially promising, as previous research showed that favorable short-term outcomes are often not sustained in the long-term [44,47,49,55]. 

With respect to generic and weight-related QoL of the adolescents and young adults, significant improvements over time could be observed. Especially regarding weight-related QoL, we observed large improvements from baseline to all follow-ups. In total, 75.6% of the participants reported higher weight-related QoL compared to the baseline. Similar observations were made for QoL concerning social functioning, and school functioning. Again, between 64.7% and 57.5% of the participants reported higher generic QoL as compared to baseline. These results indicate that comprehensive inpatient weight loss programs can be successful not only in reducing excessive weight, but also in increasing psychosocial well-being. Although effects of comprehensive life-style interventions on the participants’ QoL have only been rarely studied [47,52], mainly positive effects on QoL are reported [36,45,49,52,53]. As compared to changes in generic and disease-specific QoL reported in the previously mentioned studies among children and adolescents, our observations are more promising. However, with respect to the examined age-group of older adolescents and young adults, we are only aware of one study including QoL as outcome measure [37]. Unfortunately, results for the time effect were not reported. 

Our data suggest that weight loss corresponds to improvements of QoL, but we only observed significant correlations between the successful maintenance of the participants’ weight losses at short-term follow-up and corresponding changes in their weight-related, as well as emotional-related QoL. Similarly, their successfully sustained weight reduction one year after the end of the intervention was significantly associated with corresponding changes in their weight-related QoL. It might be concluded, that weight loss was, at least partly, associated with improvements in QoL, especially regarding the weight-related dimension. However, no final conclusion can be drawn. In literature, findings on the association between weight status and QoL are divergent and evidence is limited [34]. While similar observations reported in previous research [35,36,45] indicate a positive association, recent meta-analyses did not find such an association [34,53]. The lower correspondence between weight loss and generic QoL might be due to the importance of other variables in this context. Murray and colleagues [53] assumed children and adolescents with obesity reported reduced QoL due to social consequences of obesity, rather than actual weight. 

### 4.3. Strengths and Limitations

The results of the study have to be discussed in light of the strengths and limitations of our research. On the side of limitations, several aspects should be considered: First, although we invested enormous time and effort, the high amount of incomplete or missing data over time (between 30% and 40%, see Figure 1) jeopardizes the validity of the results. High attrition rates are not uncommon in this age and condition group [55,56], but contrary to previous research [36], we observed no systematic drop-out related to baseline BMI-SDS. Furthermore, results of ITT could be confirmed by PPA without the imputation of missing values. Similarly, results were mostly confirmed in the sensitivity analyses. They reported slight deviations regarding school functioning, referring only to two clinics and revealing no clear direction. However, future research might benefit from home visits that have shown to contribute to the reduction of attrition rates [57]. Second, we had to refer our weight-related analyses to a mixture of objectively assessed and self-reported weight data. Self-reported data might be biased with respect to actual weight loss (e.g., [58]), but we found high correlations between the two data sources. Furthermore, we replicated our analyses based on the objective weight data (*n* = 101) and yielded the same results (data not shown). In addition, since we concentrated on the achieved weight loss comparing two active treatment arms, one would not expect a selection bias in one or the other intervention group. Third, we only realized a cluster-randomization of the participants which was based on their dates of arrival in the clinic, in order to avoid spill-over effects. Future research should concentrate on complete randomization of the patients. Fourth, further studies should also consider the influence of gender and educational background on the effectiveness of life-style interventions. Our study was not powered to analyze gender-specific effects or even the interaction between gender and maturity status.

Several strengths of our study should be pointed out. First, to our knowledge, our study was the first investigating treatment effects of age-specific interventions for adolescents and young adults with obesity not only in regard of weight loss, but also considering changes in QoL. Second, in our study, we compared two comprehensive treatments that only differed in the contents and the group composition of the CBT sessions; therefore, controlling for a number of confounding variables. The analysis within the homogenous age-group of emerging adults should be pointed out especially. Furthermore, we conducted a 1-year follow-up in a controlled design. Finally, we measured weight and height at every time point and used validated questionnaires for the assessment of QoL.

## 5. Clinical Implications

Several clinical implications can be pointed out. We found that an age-sensitive approach for adolescents and young adults with respect to training contents and group composition is not superior to an approach that addresses a wider age-range in terms of weight loss and QoL. In general, our results suggest that available cognitive-behavioral interventions mainly developed for younger children and adolescents are also applicable and efficient for older adolescents and young adults, at least in the context of a comprehensive inpatient intervention. Those interventions not only led to clinically significant weight reductions, but also to clinically relevant improvements in weight-related and generic QoL. Especially promising is that said effects were still present one year after the end of the intervention. So far, older adolescents and young adults have been neglected in research and in that respect, our study expands previous research. However, further research is warranted to examine the generalizability of our results to different settings. 

## Figures and Tables

**Table 1:** Program contents.

Lessons	Topic	Contents
1	Building up motivation to life-style changes	Becoming acquainted; consequences of obesity; pros and cons of changing behavior; conveying positive prospects of moderate weight loss
2	Goalsetting	Etiology of obesity; key of success: defining adequate goals, self-monitoring, commitment to behavior change; diet and physical activity; introduction of goal-diary as weekly “home-work”
3	Diet	Vicious circle of dieting and weight-cycling; questioning of common diet myths
4	Eating behavior	Self-reflection regarding eating behavior; introduction of self-control strategies (so-called “Fit-Tricks”) and building up of alternative behavior
5	Dealing with stress and everyday problems	Identifying individual triggers for eating; introduction of coping-strategies; education on advantages of regular exercising
6	Social competence	Harassment and self-assertion: ego-strength as resource, reframing stigma experiences for self-esteem stabilization
7	Parental and social support	Sharing responsibility and task distribution (parents vs. child); autonomy; asking for parental/social support; letter to the parents
8	Profession and school	Vocational choice, application, job interview; critical questions with respect to weight status; training of social competence
9	Relapse prevention	Immunization by mental anticipation of unfavorable weight course; preventing relapse; summing up of learned strategies; long-term target tracking (goal-diary, apps); farewell

**Table 2:** Sample characteristics of the intervention group and the group receiving treatment as usual at baseline.

		IG (*n* = 141)		TAU (*n* = 125)	
		*M*	*SD*	*M*	*SD*
Age (years, range: 16–21) *		17.64	1.10	17.33	1.12
BMI-SDS ^a^		2.90	0.54	2.93	0.45
Perceived financial security (range: 0–6)		4.85	1.17	5.05	1.17
		*n*	%	*n*	%
Sex	female	97	68.8	77	61.6
	male	44	31.2	48	38.4
BMI classification ^a^	obese	20	14.2	13	10.4
	severely obese	118	83.7	107	85.6
Education ^b^	special school	6	4.5	8	6.7
	general/secondary/vocational school	97	72.9	78	65
	schools leading to the European Baccalaureate	30	22.6	34	28.3

Note: M = mean; SD = standard deviation; IG = intervention group; TAU = treatment as usual; BMI-SDS = body mass index standard deviation score; BMI = body mass index. ^a^ Conversion in BMI-SDS and weight classification in obese = BMI > 97th percentile; severely obese = BMI > 99.5th percentile (*n*_TAU_ = 120, *n*_IG_ = 138) in line with national recommendations [38]. ^b^ Type of school the participants currently attend or last attended (*n*_TAU_ = 120, *n*_IG_ = 133). * *p* < 0.05.

**Table 3:** Results of analyses of covariance for short- and long-term effects of treatment allocation.

		6-Month Follow-Up		12-Month Follow-Up	
Variable	ITT/PPA	ANCOVA	ANCOVA	ANCOVA	ANCOVA
		(main effect group)	(covariate)	(main effect group)	(covariate)
BMI-SDS	ITT	*MD* = 0.00,	*B* = 0.83, *SE* = 0.05,	*MD* = −0.07,	*B* = 0.75, *SE* = 0.05,
		*F* (1, 263) = 0.00,	*F* (1, 263) = 262.42,	*F* (1, 263) = 1.74,	*F* (1, 263) = 193.12,
		*p* = 0.962, *partial η^2^* < 0.001	*p* < 0.001, *partial η^2^* = 0.499	*p* = 0.188, *partial η^2^* = 0.007	*p* < 0.001, *partial η^2^* = 0.423
	PPA	*MD* = −0.1,	*B* = 1.09, *SE* = 0.06,	*MD* = −0.12,	*B* = 1.01, *SE* = 0.07,
		*F* (1, 163) = 2.25,	*F* (1, 163) = 305.89,	*F* (1, 163) = 3.17,	*F* (1, 163) = 236.43,
		*p* = 0.136, *partial η^2^* = 0.014	*p* < 0.001, *partial η^2^* = 0.652	*p* = 0.077, *partial η^2^* = 0.019	*p* < 0.001, *partial η^2^* = 0.592
GW-QL-KJ	ITT	*MD* = 0.79,	*B* = 0.40, *SE* = 0.05,	*MD* = 1.96,	*B* = 0.31, *SE* = 0.05,
		*F* (1, 263) = 0.13,	*F* (1, 263) = 52.95,	*F* (1, 263) = 0.93,	*F* (1, 263) = 36.08,
		*p* = 0.715, *partial η^2^* = 0.001	*p* < 0.001, *partial η^2^* = 0.168	*p* = 0.337, *partial η^2^* = 0.004	*p* < 0.001, *partial η^2^* = 0.121
	PPA	*MD* = 1.50,	*B* = 0.66, *SE* = 0.09,	*MD* = 1.06,	*B* = 0.58, *SE* = 0.09,
		*F* (1, 136) = 0.19,	*F* (1, 136) = 57.81,	*F* (1, 136) = 1.00,	*F* (1, 136) = 45.39,
		*p* = 0.660, *partial η^2^* = 0.001	*p* < 0.001, *partial η^2^* = 0.298	*p* = 0.756, *partial η^2^* = 0.001	*p* < 0.001, *partial η^2^* = 0.250
Peds-QL: emotional functioning	ITT	*MD* = 2.24,	*B* = 0.34, *SE* = 0.05,	*MD* = 0.83,	*B* = 0.30, *SE* = 0.05,
		*F* (1, 263) = 1.06,	*F* (1, 263) = 44.32,	*F* (1, 263) = 0.18,	*F* (1, 263) = 44.53,
		*p* = 0.303, *partial η^2^* = 0.004	*p* < 0.001, *partial η^2^* = 0.144	*p* = 0.668, *partial η^2^* = 0.001	*p* < 0.001, *partial η^2^* = 0.145
	PPA	*MD* = −1.94,	*B* = 0.58, *SE* = 0.08,	*MD* = −2.63,	*B* = 0.53, *SE* = 0.08,
		*F* (1, 135) = 0.32,	*F* (1, 135) = 54.53,	*F* (1, 135) = 0.56,	*F* (1, 135) = 43.14,
		*p* = 0.575, *partial η^2^* = 0.002	*p* < 0.001, *partial η^2^* = 0.288	*p* = 0.458, *partial η^2^* = 0.004	*p* < 0.001, *partial η^2^* = 0.242
Peds-QL: social functioning	ITT	*MD* = 1.26,	*B* = 0.33, *SE* = 0.04,	*MD* = 1.81,	*B* = 0.26, *SE* = 0.04,
		*F* (1, 263) = 0.52,	*F* (1, 263) = 78.92,	*F* (1, 263) = 0.16,	*F* (1, 263) = 49.89,
		*p* = 0.472, *partial η^2^* = 0.002	*p* < 0.001, *partial η^2^* = 0.231	*p* = 0.282, *partial η^2^* = 0.004	*p* < 0.001, *partial η^2^* = 0.159
	PPA	*MD* = 1.06,	*B* = 0.52, *SE* = 0.06,	*MD* = 0.76,	*B* = 0.41, *SE* = 0.06,
		*F* (1, 135) = 0.14,	*F* (1, 135) = 77.05,	*F* (1, 135) = 0.06,	*F* (1, 135) = 41.76,
		*p* = 0.709, *partial η^2^* = 0.001	*p* < 0.001, *partial η^2^* = 0.363	*p* = 0.805, *partial η^2^* < 0.001	*p* < 0.001, *partial η^2^* = 0.236
Peds-QL: school functioning	ITT	*MD* = −3.33,	*B* = 0.39, *SE* = 0.05,	*MD* = 0.26,	*B* = 0.33, *SE* = 0.48,
		*F* (1, 263) = 3.05,	*F* (1, 263) = 66.75,	*F* (1, 263) = 0.02,	*F* (1, 263) = 50.94,
		*p* = 0.082, *partial η^2^* = 0.011	*p* < 0.001, *partial η^2^* = 0.202	*p* = 0.890, *partial η^2^* < 0.001	*p* < 0.001, *partial η^2^* = 0.162
	PPA	*MD* = −6.04,	*B* = 0.63, *SE* = 0.08,	*MD* = −3.33,	*B* = 0.59, *SE* = 0.08,
		*F* (1, 135) = 3.71,	*F* (1, 135) = 66.97,	*F* (1, 135) = 1.09,	*F* (1, 135) = 57.32,
		*p* = 0.056, *partial η^2^* = 0.027	*p* < 0.001, *partial η^2^* = 0.332	*p* = 0.300, *partial η^2^* = 0.008	*p* < 0.001, *partial η^2^* = 0.298

Note: BMI-SDS = body mass index standard deviation score; GW-LQ-KJ = weight-specific Quality of Life questionnaire for overweight and obese children and adolescents; Peds-QL = Pediatric Quality of Life Inventory; ITT = intention-to-treat analysis; PPA = per-protocol analysis; ANCOVA = analysis of covariance; MD = mean difference; B = unstandardized beta; SE = standard error.

**Table 4:** Means and standard deviation scores for the intention-to-treat analyses and per-protocol analyses. The measurements were assessed for the intervention group and the group receiving treatment as usual at baseline, at post treatment and at 6-Month and 12-Month follow-ups.

	ITT				PPA			
	IG		TAU		IG		TAU	
	*M*	*SD*	*M*	*SD*	*M*	*SD*	*M*	*SD*
BMI-SDS								
T1	2.90	0.54	2.93	0.45	2.83	0.57	2.89	0.48
T2	2.58	0.59	2.61	0.48	2.51	0.63	2.59	0.53
T3	2.38	0.62	2.40	0.55	2.40	0.77	2.36	0.64
T4	2.48	0.58	2.43	0.58	2.46	0.71	2.40	0.68
GW-LQ-KJ								
T1	41.38	21.26	43.49	18.52	42.87	21.16	45.56	18.19
T2	54.96	19.12	60.13	21.03	56.59	20.32	64.48	21.99
T3	60.06	19.37	61.69	19.25	58.99	24.43	62.27	23.32
T4	55.36	18.28	57.96	16.77	55.49	24.51	58.11	21.24
Peds-QL: emotional								
T1	57.65	22.34	62.09	20.47	55.47	23.04	64.93	20.26
T2	60.29	22.34	65.11	19.67	60.35	24.70	67.69	19.69
T3	63.00	18.33	66.72	19.62	63.21	23.22	66.76	23.65
T4	60.27	15.53	62.44	18.46	60.39	21.81	62.75	24.39
Peds-QL: social								
T1	70.89	24.04	74.01	22.18	69.56	24.38	73.03	23.91
T2	77.79	21.29	83.16	17.37	77.52	21.23	83.73	20.11
T3	81.12	15.46	83.42	16.87	79.53	19.76	82.39	21.68
T4	79.01	14.11	81.62	15.67	78.58	19.42	80.77	21.46
Peds-QL: school								
T1	66.69	19.72	69.43	20.54	66.25	17.95	71.18	22.42
T2	74.99	16.90	77.58	17.40	74.92	17.81	80.37	18.62
T3	73.71	14.48	71.44	20.03	73.36	19.00	70.42	24.98
T4	70.24	14.10	71.41	19.12	71.32	19.38	70.92	24.51

Note: M = mean; SD = standard deviation; ITT = intention-to-treat; PPA = per-protocol analysis; IG = intervention group; TAU = treatment as usual; T1 = baseline; T2 = post treatment; T3 = 6-month follow-up; T4 = 12-month follow-up; BMI-SDS = body mass index standard deviation score; GW-LQ-KJ = weight-specific Quality of Life questionnaire for overweight and obese children and adolescents; Peds-QL = Pediatric Quality of Life Inventory. ITT: *n*_IG_ = 141, *n*_TAU_ = 125. PPA: BMI-SDS: *n*_IG_ = 84, *n*_TAU_ = 82; GW-LQ-KJ: *n*_IG_ = 67, *n*_TAU_ = 72; Peds-QL: *n*_IG_ = 67, *n*_TAU_ = 71.
